# Mitochondrial Respiration Defects in Single-Ventricle Congenital Heart Disease

**DOI:** 10.3389/fcvm.2021.734388

**Published:** 2021-09-23

**Authors:** Xinxiu Xu, Jiuann-Huey Ivy Lin, Abha S. Bais, Michael John Reynolds, Tuantuan Tan, George C. Gabriel, Zoie Kondos, Xiaoqin Liu, Sruti S. Shiva, Cecilia W. Lo

**Affiliations:** ^1^Department of Developmental Biology, School of Medicine, University of Pittsburgh, Pittsburgh, PA, United States; ^2^Department of Critical Care Medicine, School of Medicine, University of Pittsburgh, Pittsburgh, PA, United States; ^3^School of Medicine, Pittsburgh Heart, Lung, Vascular Medicine Institute, University of Pittsburgh, Pittsburgh, PA, United States; ^4^Department of Pharmacology and Chemical Biology, School of Medicine, University of Pittsburgh, Pittsburgh, PA, United States

**Keywords:** oxygen consumption rate (OCR), single ventricle congenital heart disease (SV-CHD), peripheral blood mononuclear cells (PBMC), heart failure, Fontan, reserve OCR, maximal respiratory capacity, hypoplastic left heart syndrome (HLHS)

## Abstract

**Background:** Congenital heart disease (CHD) with single-ventricle (SV) physiology is now survivable with a three-stage surgical course ending with Fontan palliation. However, 10-year transplant-free survival remains at 39–50%, with ventricular dysfunction progressing to heart failure (HF) being a common sequela. For SV-CHD patients who develop HF, undergoing the surgical course would not be helpful and could even be detrimental. As HF risk cannot be predicted and metabolic defects have been observed in *Ohia* SV-CHD mice, we hypothesized that respiratory defects in peripheral blood mononuclear cells (PBMCs) may allow HF risk stratification in SV-CHD.

**Methods:** SV-CHD (*n* = 20), biventricular CHD (BV-CHD; *n* = 16), or healthy control subjects (*n* = 22) were recruited, and PBMC oxygen consumption rate (OCR) was measured using the Seahorse Analyzer. Respiration was similarly measured in *Ohia* mouse heart tissue.

**Results:** Post-Fontan SV-CHD patients with HF showed higher maximal respiratory capacity (p = 0.004) and respiratory reserve (*p* < 0.0001), parameters important for cell stress adaptation, while the opposite was found for those without HF (reserve *p* = 0.037; maximal *p* = 0.05). This was observed in comparison to BV-CHD or healthy controls. However, respiration did not differ between SV patients pre- and post-Fontan or between pre- or post-Fontan SV-CHD patients and BV-CHD. Reminiscent of these findings, heart tissue from *Ohia* mice with SV-CHD also showed higher OCR, while those without CHD showed lower OCR.

**Conclusion:** Elevated mitochondrial respiration in PBMCs is correlated with HF in post-Fontan SV-CHD, suggesting that PBMC respiration may have utility for prognosticating HF risk in SV-CHD. Whether elevated respiration may reflect maladaptation to altered hemodynamics in SV-CHD warrants further investigation.

## Introduction

Hypoplastic left heart syndrome (HLHS) is a critical congenital heart disease (CHD) characterized by hypoplasia of left-sided structures of the heart, including the ascending aorta, left ventricle (LV), and aortic and mitral valves. As there is only a single pumping chamber in HLHS [the right ventricle (RV)], it is a type of single-ventricle (SV) CHD. While HLHS and other SV-CHDs were previously uniformly fatal, it is now survivable with a three-stage surgical palliation that includes the final Fontan surgery. Together, this creates an SV circulation with venous blood routed directly to the lungs by gravity, while the single pumping chamber (LV or RV) serves as the systemic chamber pumping blood to the rest of the body (1).

While such SV physiology (2) allows most SV-CHD patients to survive, 10-year transplant-free survival for HLHS patients stands at only 39–50%. This high morbidity/mortality is largely due to ventricular dysfunction and heart failure (HF) (3) for which heart transplantation remains the ultimate therapy. Therapies developed for adult HF have been ineffective for SV-CHD (3). As it is not possible to predict which SV-CHD patients will have HF, it has not been possible to prioritize SV-CHD patients for early heart transplantation. For SV-CHD patients who will develop HF, undergoing the palliative surgical course would not be helpful and could even be detrimental.

Our previous study of a mouse model of HLHS (4) uncovered mitochondrial abnormalities that suggested metabolic disturbance contributing to the pathogenesis of HLHS. Consistent with this, transcriptome profiling of the HLHS mouse heart tissue yielded metabolic pathways among the top pathways impacted (4). Supporting clinical evidence has come from a recent study showing that serum isolated from SV-CHD patients can induce pathological changes in neonatal rat cardiomyocytes (5). Transcriptome profiling of the treated cardiomyocytes showed that 23% of the genes impacted were related to metabolic processes (5). Another study of adults at early stages of HF showed that their peripheral blood mononuclear cells (PBMCs) had mitochondrial respiration defects in association with inflammation and oxidative stress (6).

Based on these previous findings, in the present pilot study, we investigated the hypothesis that respiratory parameters in PBMC may have prognostic value for assessing HF risk in SV-CHD. For this study, we recruited SV-CHD patients pre- vs. post-Fontan completion and also age-matched BV-CHD patients and control subjects.

## Methods

### Patient Recruitment

CHD patients and healthy control subjects were recruited from the Children's Hospital of Pittsburgh of University of Pittsburgh Medical Center (UPMC) under a human study protocol approved by the University of Pittsburgh Institutional Review Board. Informed consent was obtained, and for infants and minors, informed consent was obtained from the parent or legal guardian. Recruitment criteria comprised subjects with complex SV-CHD or BV-CHD. For SV-CHD, this included HLHS, critical aortic stenosis, pulmonary atresia with intact ventricular septum, double outlet RV with mitral atresia, and unbalanced atrioventricular septal defect with total or partial anomalous pulmonary venous return, while BV-CHD included Tetralogy of Fallot, transposition of the great arteries, Ebstein's anomaly, aortic arch hypoplasia, aortic stenosis, ventricular septal defect, and pulmonary stenosis. Three pediatric dilated cardiomyopathy (DCM) patients with HF were recruited as BV-HF disease controls, two having received a heart transplant and the third listed for transplant and having received an interim ventricular assist device. Patient demographics are summarized in Table 1, Supplementary Spreadsheet S1.

**Table 1:** Demographic and clinical outcome summary.

	**≥10 years of age**				**≤1 year of age**		
	**Control**	**BV**	**SV non-HF**	**SV HF**	**BV**	**SV non-HF**	**SV HF**
No. subjects	22	5	4	4	**11**	11	1
Male (%)	9 (59%)	2 (40%)	4 (100%)	3(75%)	8 (73%)	4 (36.3%)	1 (100%)
Mean age (years) ^@^	29 ± 1.4	25.8 ± 8.79	23.75 ± 10.5	17.3 ± 4.92	0.28 ± 0.34	0.38 ± 0.31	0.096
Caucasian		4 (80%)	4 (100%)	2 (50%)	8 (72.7%)	8 (72.7%)	1 (100%)
African American		1 (20%)	0	2 (50%)	3 (27.3%)	3 (27.3%)	
Mean BMI (kg/m^2^)		25.2 ± 6.06	25.7 ± 8.09	20.9 ± 2.56	13.9 ± 2.36	15.3 ± 3.82	12.5
Age of Fontan		NA	2.75 ± 0.5	5.5 ± 3.11	NA	NA	NA
Mean Fontan Years		NA	21 ± 10.1	11.8 ± 4.99	NA	NA	NA
${\text{SpO}}_{2}^{\text{\$}}$		98.8 ± 0.84	91.5 ± 7.14	92 ± 4.6	98.2 ± 2.7	82.54 ± 6	83
NYHA Class^#^^*^		1.6 ± 0.55	1.25 ± 0.5	2.75 ± 0.5	1.63 ± 0.9	2.18 ± 0.87	3
**Systemic ventricle**							
Dominant RV			3 (75%)	4 (100%)		8 (72.7%)	1 (100%)
Dominant LV			1 (25%)	0		3 (27.3%)	

@ *Mean age (years): ≥10 years old, Control vs. SV HF, p = 0.0003*.

$ *SpO_2_: BV vs. SV-HF ≥10 years old, p = 0.014; BV vs. SV non-HF ≤ 1-year old, p < 0.0001*.

# *NYHA (26) Functional Classification was used to classify patients' heart failure*.

* *NYHA: BV vs. SV non-HF ≥10 years old, p = 0.36; BV vs. SV HF, ≥10 years old, p = 0.014; SV non-HF vs. SV HF, ≥10 years old, p = 0.005*.

*BMI, body mass index; BV, biventricular; HF, heart failure; LV, left ventricle; NYHA, New York Heart Association; RV, right ventricle; SV, single ventricle*.

### Peripheral Blood Mononuclear Cell Isolation and Measurement of Oxygen Consumption Rate

Whole blood was used to recover fresh PBMCs using standard protocol with Ficoll-Paque PLUS from Pharmacia Biotech (or a similar separation medium). After centrifugation, PBMCs at the interphase were collected, washed twice, and resuspended in RPMI 1640 with 10% fetal bovine serum, and the cells were counted. For measurement of oxygen consumption rate (OCR) and extracellular acidification rate (ECAR), 20,000 PBMCs were seeded into each well of a Seahorse XFe96 plate. OCR and ECAR were determined using the Seahorse XF Cell Mito Stress Kit (Agilent). Basal respiration was measured in unstimulated cells. Afterward, oligomycin (1 μM) was added to quantify respiration coupled to ATP production and proton leak followed by carbonyl cyanide-4-(trifluoromethoxy)-phenylhydrazone (FCCP; 1 μM) injection to assess maximal cellular respiration (respiratory capacity). Finally, antimycin A (1 μM) and rotenone (1 μM) were used to assess non-mitochondrial respiration. For mouse heart tissue, OCR was obtained for the quantification of basal respiration. For this analysis, 2-mm^2^ pieces of heart tissue were plated into individual wells of a Seahorse XF24 microplate, and OCR was measured using the same Seahorse XF Cell Mito Stress Kit without stimulation during two cycles of measurement.

## Results

CHD patients with BV-CHD (*n* = 16) or SV-CHD (*n* = 20) and 22 healthy controls without CHD were recruited with informed consent, and blood samples were obtained for PBMC isolation. Among the 20 SV-CHD patients, 16 had HLHS with dominant/systemic RV, and four had dominant/systemic LV (Table 1). Twelve of the SV patients were <1 year old, while eight were ≥10 years old, all having completed the Fontan surgery (Table 1). Of the BV patients, five were ≥10 years old, and 11 were <1 year old. All the control subjects were ≥10 years old (Table 1).

All of the BV-CHD (*n* = 16) patients and 15 of the 20 SV-CHD patients survived heart transplant-free and were classified as having a favorable clinical outcome without HF. Among the SV patients, five HLHS patients had an unfavorable cardiac outcome related to HF, one having died from acute HF, and four exhibiting cardiac death. The latter was indicated by having had a heart transplant (*n* = 3) or having been listed for heart transplant for decompensated HF (*n* = 1). Using the New York Heart Association (NYHA) HF classification, the four HLHS patients ≥10 years old with HF had significantly higher or worse NYHA scores than either the BV-CHD patients (*p* = 0.014) or the SV-CHD patients with favorable outcome (*p* = 0.005) (Table 1).

### Mitochondrial Respiration in Peripheral Blood Mononuclear Cells of SV-CHD and BV-CHD Patients

PBMCs isolated from the blood of patients and controls were analyzed for OCR using the Seahorse Analyzer. Measurements of the different mitochondrial respiratory parameters were obtained by assessing the changes in OCR with the addition of different inhibitors of respiration. No significant difference in OCR was observed among the control subjects stratified by age, although a trend for lower OCR was observed in patients >40 years old (Supplementary Figure S1A). This is consistent with a previous observation of lower mitochondrial respiration in platelets from older subjects (7). Hence, we excluded OCR measurements from subjects >40 years of age and used only the 22 control subjects between 10 and 40 years of age (Supplementary Figure S1A).

**Figure 1:**
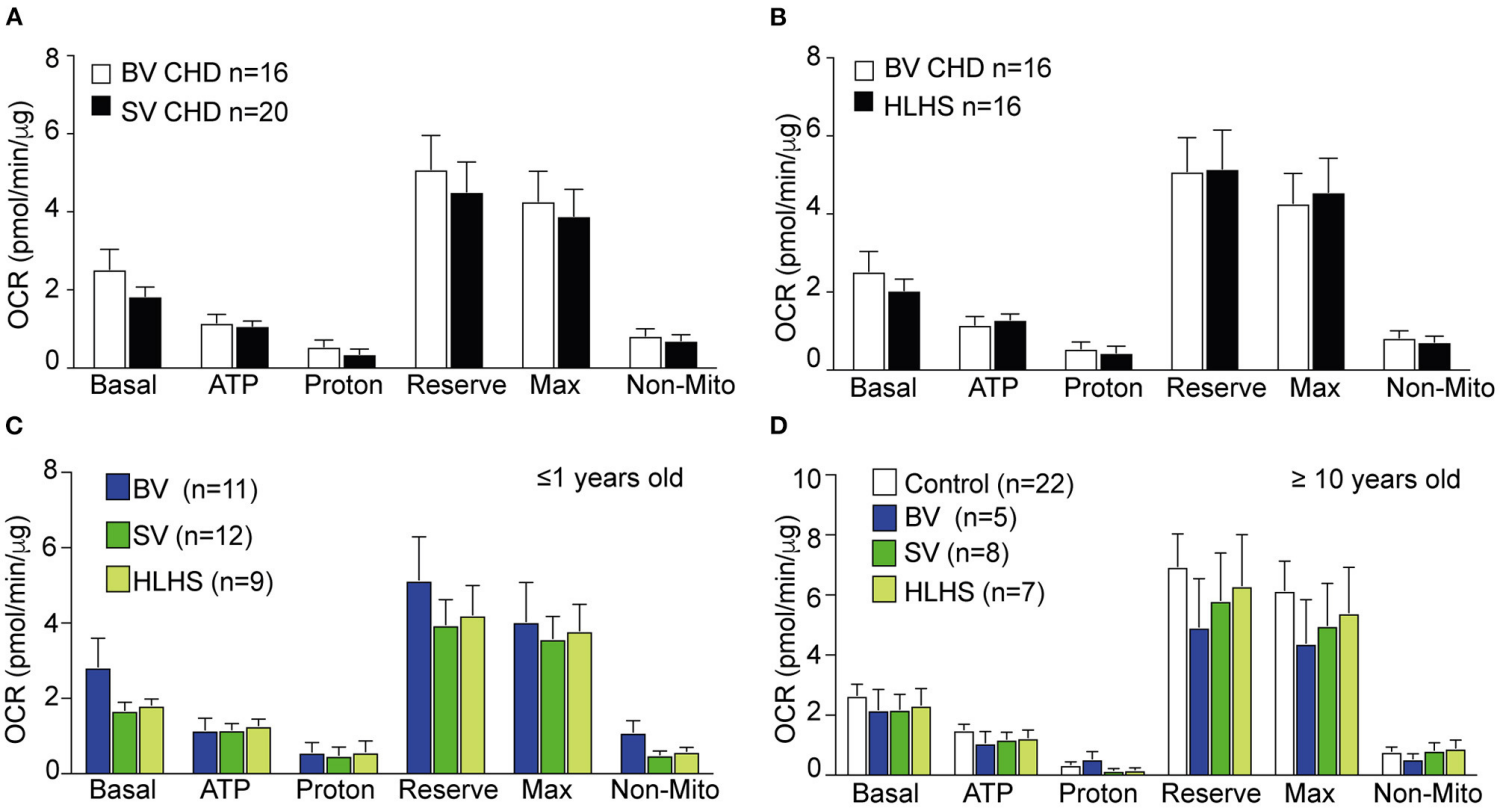
Comparison of mitochondrial respiration in the PBMCs of CHD patients stratified by lesion type, age, and Fontan status. **(A,B)** There was no difference in any of the respiratory parameters in OCR of PBMCs of BV-CHD vs. SV-CHD and between BV-CHD and HLHS. **(C)** No significant difference was observed in the parameters of mitochondrial respiration among BV-CHD, SV-CHD, and HLHS patients ≤1 year old. **(D)** No significant difference in mitochondrial respiration was observed among control, BV-CHD, SV-CHD, and HLHS subjects ≥10 years old. Bar graphs show mean ± SEM with one-way ANOVA. Subjects' numbers were indicated in the graphs. BV, biventricular; CHD, congenital heart disease; HLHS, hypoplastic left heart syndrome; OCR, oxygen consumption rate; PBMC, peripheral blood mononuclear cell; SV, single ventricle.

Comparisons between the SV-CHD and BV-CHD patients showed no difference in mitochondrial respiration (Figure 1A, Supplementary Figure S1B). A similar comparison of the SV-HLHS vs. BV-CHD patients also showed no difference (Figure 1B, Supplementary Figure S1C). We further examined the BV and SV patients grouped by age, either <1 year old or >10 years old (Supplementary Table S1). This also showed no difference for any mitochondrial respiratory parameter (Figures 1C,D). For individuals ≥10 years old, no difference was observed for any of the CHD groups when compared with healthy controls (Figure 1D). We further examined basal glycolysis with the measurement of the ECAR, and this also showed no difference between the SV-CHD and BV-CHD patients (Supplementary Figure S1D).

### Respiration in Pre- and Post-fontan SV-CHD Patients

**Figure 2:**
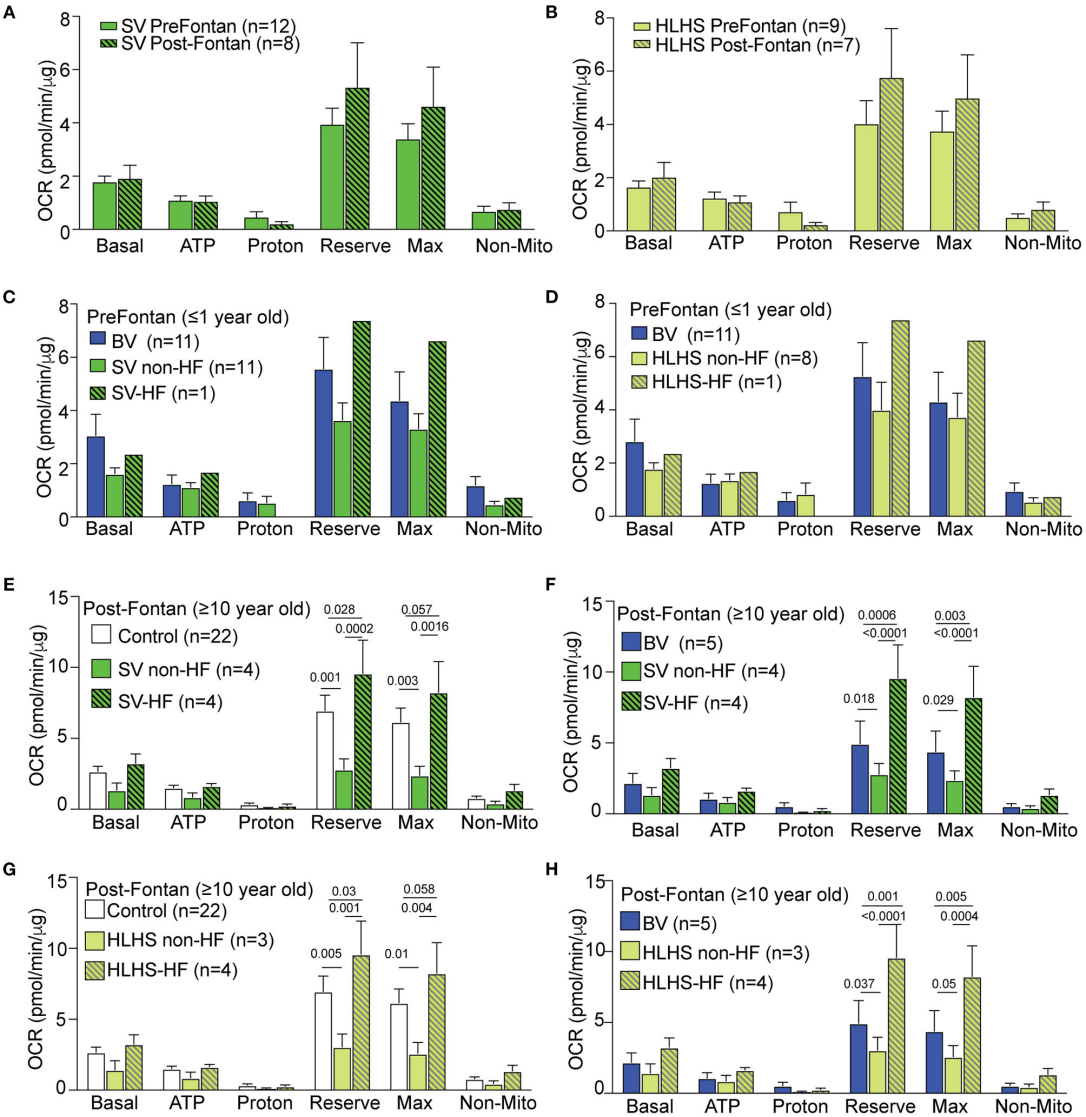
Mitochondrial respiration in SV-CHD patients stratified by Fontan palliation and HF status. **(A,B)** No significant difference was observed for respiration in pre- vs. post-Fontan SV and HLHS patient groups. **(C,D)** Mitochondrial respiration in the PMBCs of pre-Fontan SV **(C)** or HLHS patients **(D)** ≤1 year old as compared to age-matched BV patients. **(E,F)** Mitochondrial respiration in the PBMCs of SV-CHD patients ≥10 years old with and without HF vs. age-matched controls **(E)** and BV-CHD patients **(F)**. **(G,H)** Mitochondrial respiration in the PBMCs of HLHS-CHD patients ≥10 years old with and without HF vs. age-matched controls **(G)** and BV-CHD patients **(H)**. Mean ± SEM with one-way ANOVA test. Subjects' numbers are indicated in the legend for the graphs. BV, biventricular; CHD, congenital heart disease; HF, heart failure; HLHS, hypoplastic left heart syndrome; OCR, oxygen consumption rate; PBMC, peripheral blood mononuclear cell; SV, single ventricle.

Comparison of the pre-Fontan (<1 year old) vs. post-Fontan (>10 years old) SV or HLHS patients showed no difference in mitochondrial respiration (Figures 2A,B). ECAR data also showed no difference (Supplementary Figure S1E). We further investigated the impact of HF on mitochondrial respiration in the pre- vs. post-Fontan patients. Among the 12 pre-Fontan SV patients, only one had an unfavorable outcome with sudden cardiac death. This subject showed higher respiratory maximum and reserve when compared to the SV or BV patients without HF (Figure 2C). As this patient had HLHS, comparison was also made to the eight non-failing pre-Fontan HLHS patients. This also showed higher maximal respiration and reserve capacity in the pre-Fontan HLHS patient with HF (Figure 2D). For one HLHS patient, we obtained PBMCs for OCR measurements before and after heart transplant. No change was observed in any respiratory parameter after heart transplantation, suggesting that patient intrinsic factors and not hemodynamic factors underlie the respiratory dysfunction in this HLHS patient (Supplementary Figure S1F).

### Respiration and Clinical Outcome in Post-fontan HLHS Patients

Of the eight SV-CHD patients with Fontan completion, four had HF. These patients showed a significantly higher OCR when compared to those of SV-CHD patients without HF, BV-CHD patients, or healthy controls (Figures 2E,F). These changes were observed in two related respiratory parameters, respiratory maximum and respiratory reserve. In contrast, SV-CHD patients without HF showed a significantly lower OCR when compared to SV-CHD patients with HF, BV-CHD patients, or healthy controls (Figures 2E,F).

Given that SV-CHD with systemic RV is known to have worse clinical outcome than SV-CHD patients with systemic LV (8–10), we reanalyzed the data focusing on only SV patients with HLHS (systemic RV) either with or without HF. This analysis yielded similar findings with significantly higher respiratory maximum and reserve observed in the post-Fontan HLHS patients with HF, while the opposite was observed in HLHS patients without HF (Figures 2G,H). We also examined basal glycolysis with the measurement of ECAR, but no significant difference was found between post-Fontan SV and HLHS patients either with or without HF when compared to BV-CHD patients or control subjects (Supplementary Figures S2E,F).

To further investigate whether the increase in respiration in the SV-CHD patients with HF may be attributable to HF in general, we recruited three pediatric DCM patients with HF and obtained PBMCs for a similar analysis of mitochondrial respiration. However, no significant difference was observed in the mitochondrial respiration of the DCM patients with HF vs. the BV-CHD patients without HF (Supplementary Figure S2G). We also examined medication use among the SV patients with and without HF and observed no correlation between inotropic infusion, brain natriuretic peptide (BNP) levels, or other medication use and changes in mitochondrial respiration. Similarly, no hemodynamic parameters were correlated with changes in mitochondrial respiration in the SV-CHD patients (Supplementary Spreadsheet S1).

### Mitochondrial Respiration in Heart Tissue of *Ohia* Mutant Mice

**Figure 3:**
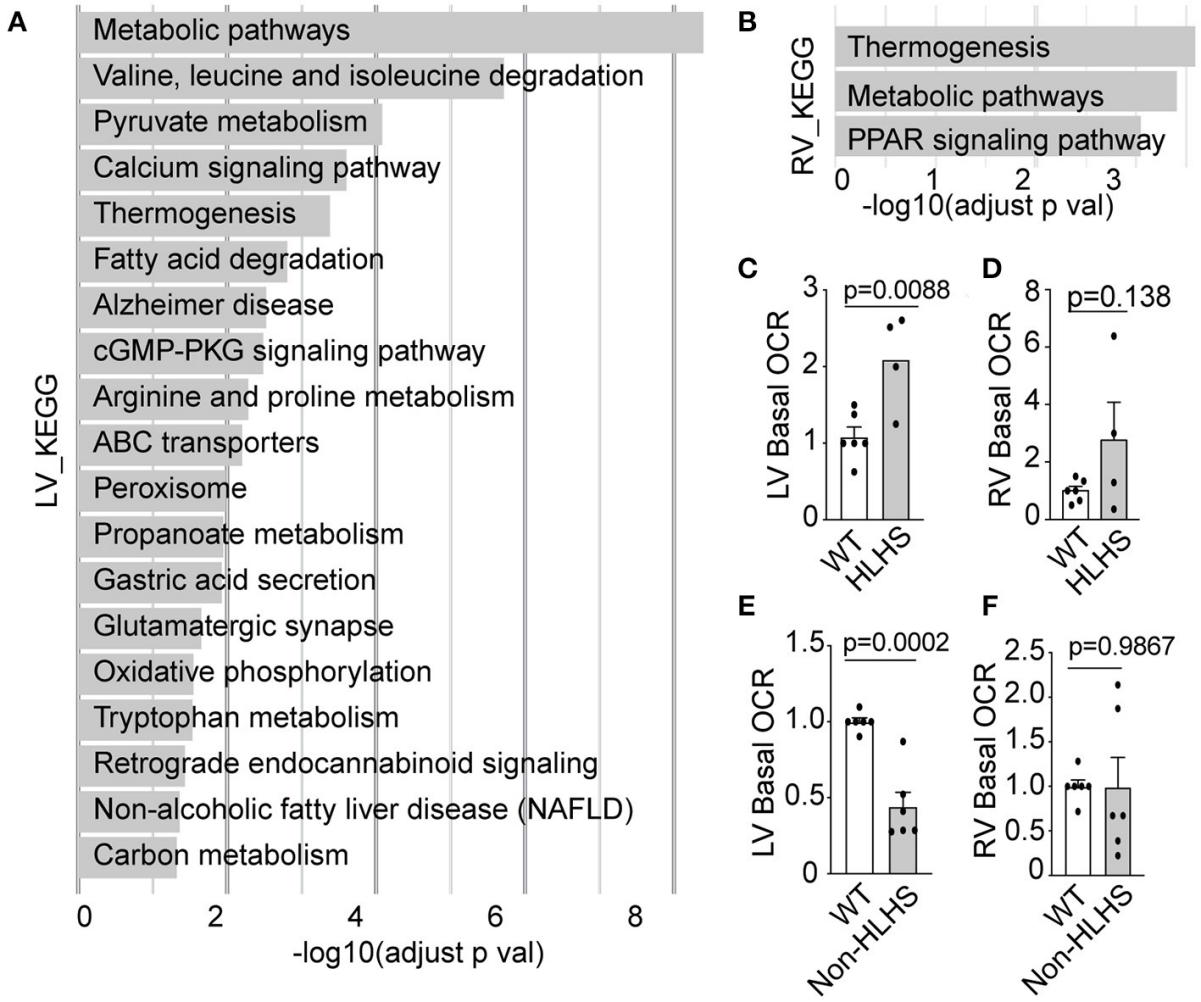
Mitochondrial respiration in *Ohia* mutant mouse heart. **(A,B)** Pathways recovered among downregulated genes at FDR 0.05 from RNA-seq analysis of HLHS-LV **(A)** or RV **(B)** tissue compared to that of WT littermate control. Pathway enrichment analysis was performed using KEGG pathway analysis with g: Profiler and FDR < 0.05. **(C–F)** Basal OCR in LV and RV heart tissue from E14.5–16.5 *Ohia* line with *Sap130/Pcdha9* mutations known to cause HLHS. This analysis was obtained using the Seahorse Analyzer. **(C,D)** Data obtained from litters comprising WT (*n* = 7) and HLHS (*n* = 4). **(E,F)** Quantitative analysis of five Sap130/Pcdha9 mutants with normal cardiac anatomy without HLHS **(E,F)** and six WT controls. Bar graphs show mean ± SEM with Student's *t*-test. Each dot represents one mouse embryo heart tissue. FDR, false discovery rate; HLHS, hypoplastic left heart syndrome; KEGG, Kyoto Encyclopedia of Genes and Genomes; LV, left ventricle; OCR, oxygen consumption rate; PPAR, peroxisome proliferator-activated receptor; RNA-seq, RNA sequencing; RV, right ventricle; WT, wild-type.

HLHS mutants from the *Ohia* mouse line were previously shown to suffer early HF with severe pericardial effusion (4) and prenatal/neonatal lethality. This was associated with mitochondria abnormalities that suggested metabolic dysfunction (4). Transcriptome profiling with RNA sequencing (RNA-seq) analysis of the HLHS heart tissue (4) recovered many metabolic pathways in the hypoplastic LV, and a few of the same metabolic pathways were also recovered in the RV but with lower fold change (Figures 3A,B, Supplementary Figures S3A,B).

Given that CHD showed incomplete penetrance in the *Ohia* mutant mice, this allowed us to further assess respiration in mice with the same HLHS-causing mutations but either with or without CHD/HLHS. Measurement of basal respiration in the heart tissue of the *Ohia* mutant mice using the Seahorse Analyzer showed elevated basal OCR in the LV tissue of HLHS mutants (Figure 3C), a result reminiscent of the elevated OCR observed in the PBMCs of HLHS/SV patients with HF. In contrast, the LV tissue of *Ohia* mice without CHD showed markedly lower basal OCR, a result that seems to parallel the lower mitochondrial respiration in the post-Fontan HLHS patients without HF (Figure 3E). However, a similar analysis of the RV tissue from *Ohia* mutant mice, whether with HLHS or without CHD, showed no significant change in respiration (Figures 3D,F). These observations point to an intrinsic mitochondrial respiration defect arising from the genetic predisposition for HLHS. Western immunoblotting showed that the respiration perturbations were not associated with altered expression of the electron transport chain (ETC) components (Supplementary Figures S3C,D).

## Discussion

Our study showed that mitochondrial respiration in PBMCs is significantly elevated in post-Fontan SV-CHD patients with poor cardiac outcome associated with HF, operationally defined as death or cardiac death with heart transplant or listing for heart transplant. This operational definition is supported by the significantly higher NYHA classification score for HF observed for HLHS/SV-CHD patients ≥10 years old identified with HF. Thus, we found that post-Fontan HLHS patients with HF exhibited higher maximal OCR and respiratory reserve, respiratory parameters often elevated in response to cell stress. In contrast, post-Fontan HLHS patients without HF showed lower maximal and reserve respiratory capacity. These findings are consistent with observations in the *Ohia* mutant mice, which also showed opposing changes in basal respiration in mutant mice with or without CHD/HLHS.

Heart tissue from *Ohia* mutant mice with HLHS showed elevated basal respiration. This is also associated with HF, shown by *in utero* echocardiography observation of poor cardiac contractility, low cardiac output, and severe pericardial effusion in the *Ohia* HLHS fetal mice (4). In contrast, genetically identical *Ohia* mutants without CHD showed reduced basal respiration but with entirely normal cardiac function. Together, these findings suggest intrinsic metabolic defects in patients with HLHS with a shared genetic etiology with their structural heart defects, a possibility supported by other studies showing mitochondrial metabolism (11) playing an important role in heart development and the regulation of cardiomyocyte differentiation (12).

Many adverse sequelae in SV-CHD patients have been attributed to the Fontan circulation with its non-pulsatile flow in the venous system (13), such as in promoting venous hypertension and hepatic congestion. However, the Fontan circulation is unlikely to account for the metabolic perturbations, as post-Fontan patients can have either hyper-elevated or reduced mitochondrial respiration. In one HLHS patient, a significant increase in mitochondrial respiration was observed before and after heart transplantation, indicating that the metabolic defects in this patient is likely intrinsic and could cause oxidative stress leading to apoptosis and HF. Supporting this possibility, induced pluripotent stem cell-derived cardiomyocytes from HLHS patients with early HF were found to have a failed antioxidant response that could exacerbate metabolic defects causing redox stress (14, 15).

While defects in mitochondrial respiration in PBMCs have been reported in patients with HF, such as in the setting of cardiomyopathies (16), this is typically associated with reduced respiratory capacity, not the hyper-elevated mitochondrial respiration observed in the post-Fontan HLHS patients with HF. Our analysis of DCM-HF patients showed no change in mitochondrial respiration when compared to that of the BV-CHD patients without HF. This would suggest that mechanistic differences may explain why existing therapies for BV-HF have been ineffective for SV-CHD. Thus, hyper-elevated mitochondrial respiration may cause increased production of reactive oxygen species (ROS) with oxidative stress ensuing and triggering apoptosis that can predispose to HF in post-Fontan patients. In contrast, reduced mitochondrial respiration in the post-Fontan SV patients without HF may keep ROS levels in check and provide cardioprotection.

The elevated maximal respiration and reserve respiratory capacity in HLHS patients with HF is not likely to be an adaptive response to the SV physiology or Fontan circulation, since only SV-CHD patients with HF showed elevated respiration. As cells usually function at only a fraction of their maximal respiratory capacity and with a correspondingly large reserve capacity (17), the elevation of these two parameters in the SV-CHD patients with HF might reflect an adaptive response to cell stress related to intrinsic mitochondrial defects. That this could be genetically encoded by the same mutations causing HLHS is suggested by observations in the *Ohia* mouse model.

While our patient assessments were conducted using PBMCs, the analysis of the *Ohia* mouse model entailed assaying OCR in heart tissue. The use of PBMCs assumes a systemic defect in mitochondrial respiration that may broadly impact a variety of different cells and tissues. This could include platelets that are cell fragments derived from megakaryocytes. Interestingly, an increase in mitochondrial reserve and maximal capacity has also been reported in the platelets of adults with pulmonary hypertension (18), a common comorbidity in CHD patients. We noted a previous study that showed that serum from SV-CHD patients (5) can cause pathological remodeling with the reactivation of fetal gene programs in neonatal rat cardiomyocytes. The expression of HF markers, such as BNP (19) and atrial natriuretic factor (20) were observed, with transcriptome profiling showing metabolic process as one of the top enriched pathways. Together, these findings suggest systemic effects impacting mitochondrial respiration in SV-CHD patients. Further studies are warranted to verify the potential utility of a simple blood test with measurement of respiration in PBMCs as a biomarker for HF risk assessment in SV-CHD patients. The possibility that platelets might also be employed for such assessments should be further investigated.

### Limitations

There are various limitations to our study, one being the complicated clinical course of older SV-CHD patients that could confound the interpretation of metabolic defects in post-Fontan patients, such as morbidity/mortality associated with protein-losing enteropathy, plastic bronchitis, or liver cirrhosis (21–24). The relatively small number of SV-CHD patients included in this study is also a limitation, largely a reflection of SV-CHD being relatively rare, comprising only 1.25% of infants with CHD (25). Due to the small cohort size, we were unable to assess the differences in mitochondrial respiration between systemic RV and systemic LV SV-CHD. Thus, a multicenter study will be needed to verify and extend the findings from this pilot study with longitudinal assessments of PBMC respiration in SV-CHD patients with dominant RV vs. LV and before and after Fontan surgery. Yet another limitation is the fact that mitochondrial respiration was investigated only in the PBMCs but not in human heart tissue. Heart tissue from patients is difficult to procure, but this can be obtained from patients undergoing heart transplant. However, changes detected in explanted heart tissue could be secondary to the HF. The lack of control pediatric PBMC measurements precluded the analysis of the CHD subjects ≤1 year of age. This limitation is partially addressed by the inclusion of age-matched pediatric BV-CHD subjects as disease controls. Finally, while our data showed a significant association between alterations in mitochondrial respiration in the PBMCs and HF risk in SV-CHD patients, establishing a causal link will require further mechanistic studies in the future. Additional studies are also needed to investigate whether PBMC respiration may be altered in other types of HF not related to CHD.

## Data Availability Statement

The datasets presented in this study can be found in online repositories. The names of the repository/repositories and accession number(s) can be found at: NCBI [accession: GSE77799].

## Ethics Statement

The studies involving human participants were reviewed and approved by University of Pittsburgh Institutional Review Board. Written informed consent to participate in this study was provided by the participants' legal guardian/next of kin. The animal study was reviewed and approved by University of Pittsburgh Institutional Animal Care and Use Committee. Written informed consent was obtained from the individual(s), and minor(s)' legal guardian/next of kin, for the publication of any potentially identifiable images or data included in this article.

## Author Contributions

CL, XX, and J-HL contributed to the study design. CL and J-HL contributed to the recruitment of subjects and human blood sample collection. XX contributed to the PBMC isolation. XX, MR, SS, and ZK contributed to the Seahorse measurement. TT, XL, and XX contributed to the mouse fetal ultrasound imaging and mouse phenotyping. GG and XX contributed to the mouse embryo dissection. AB and XX contributed to the mouse RNA-seq and data analysis. XX contributed to the statistics. CL, XX, J-HL, and SS contributed to the manuscript preparation. All authors contributed to the article and approved the submitted version.

## Funding

This work was supported by NIH grants HL132024 and HL142788 (CL), UPMC Fellows Grant (XX), and American Heart Association/ Children's Heart Foundation postdoctoral fellowship (XX) and support from Department of Critical Care Medicine (J-HL).

## Conflict of Interest

The authors declare that the research was conducted in the absence of any commercial or financial relationships that could be construed as a potential conflict of interest.

## Publisher's Note

All claims expressed in this article are solely those of the authors and do not necessarily represent those of their affiliated organizations, or those of the publisher, the editors and the reviewers. Any product that may be evaluated in this article, or claim that may be made by its manufacturer, is not guaranteed or endorsed by the publisher.
