# Supplementary material for: Mitochondrial Respiration Defects in Single-Ventricle Congenital Heart Disease
Source: Front Cardiovasc Med. 2021 Sep 23;8:734388. doi: 10.3389/fcvm.2021.734388 (PMC8494974; doi:10.3389/fcvm.2021.734388)
Supplement: Supplementary file 1 [file Data_Sheet_1.DOCX]

**Supplemental figures**

**
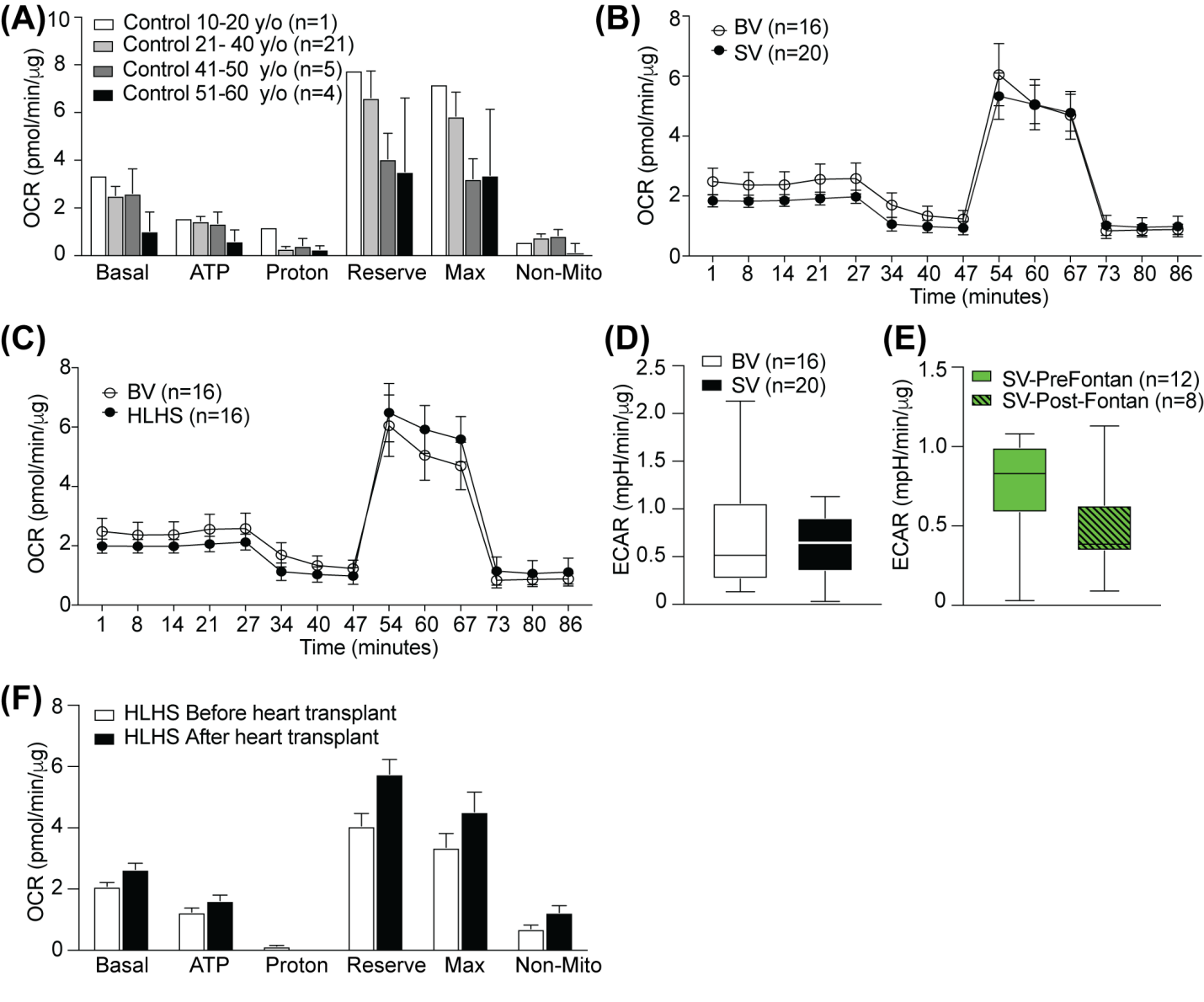
**

**Figure S1. Quantitative measurement of mitochondrial respiration and glycolysis in PBMC of control subjects and CHD patients.**

(A) Mitochondrial respiration in control subjects was noted to decline with age.

(B, C) Seahorse Analyzer profile of oxygen consumption was related to oxidative phosphorylation in the PBMCs from BV-CHD, S-CHD and HLHS patients.

(D) Basal glycolysis in BV and SV-CHD subjects.

(E) Basal glycolysis of pre-Fontan and post-Fontan SV-CHD patients.

(F) No change was observed in any respiration parameters in the PBMC of the same patient obtained before and after heart transplant.

**
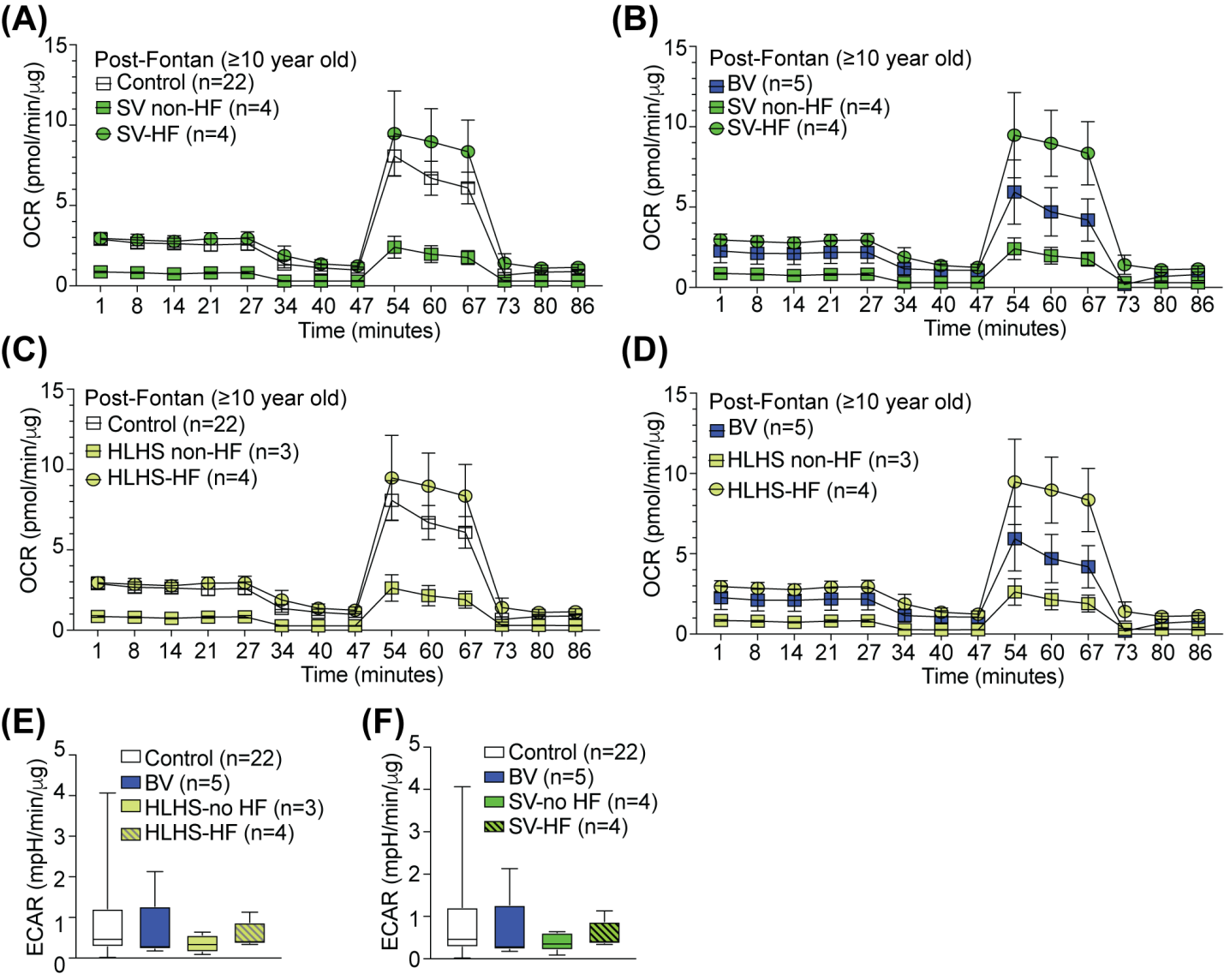
**

**Figure S2. Assessment of mitochondrial respiration and glycolysis in PBMC of post-Fontan SV-CHD with or without heart failure**

(A, B) Seahorse Analyzer profile of OCR (A) in the PBMCs of SV-CHD post-Fontan patients with and without HF (≥10 years old) was compared to that of age matched control (A) and BV-CHD patients (B).

(C, D). Seahorse Analyzer profile of OCR (A) in the PBMC of HLHS post-Fontan patients with and without HF vs. age matched control (C) and BV-CHD patients (D).

(E) Basal glycolysis in Control, BV and HLHS patients post-Fontan patients.

(F) Basal glycolysis in Control, BV and SV-CHD patients post-Fontan patients.

Box graphs (E, F) show mean value and floating bar (Min to Max), one way ANOVA test was applied. Subjects number were indicated on the graphs.


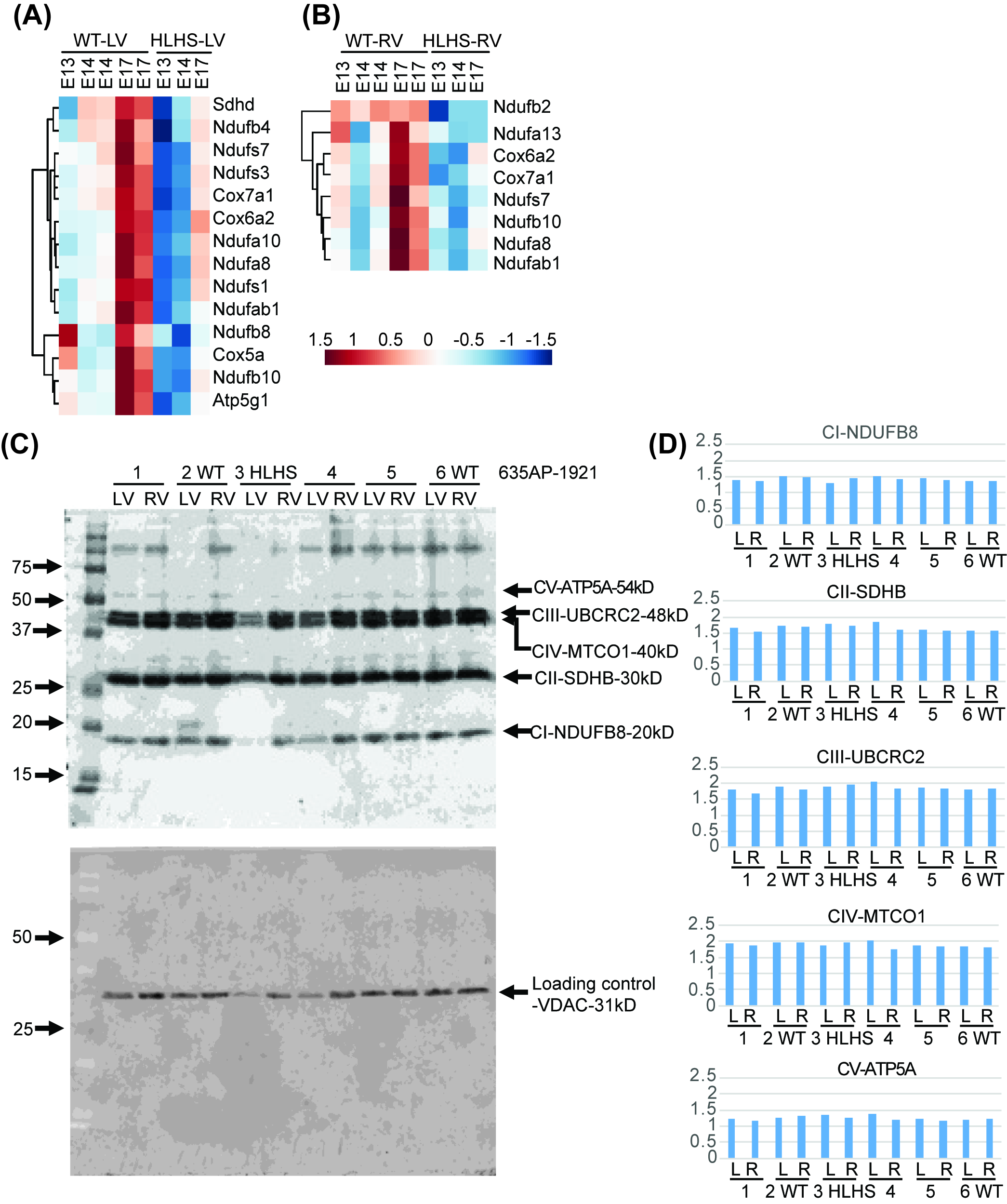


**Figure S3. RNAseq and Western blot analysis of electron transport chain components in *Ohia* mouse heart tissue**

(A, B) Heatmap of the expression of mitochondrial electron transport chain (ETC) components in the LV and RV of HLHS mutants vs. wildtype littermate controls are shown in (A) and (B) respectively.

(C, D) There was no difference in the expression of electron transport chain (ETC) proteins and voltage dependent anion channel (VDAC) by using immunoblots (C) from heart tissues of *Ohia* HLHS mutants vs. wildtype littermate controls. Quantification of the Western blots (D) showed no difference in expression between the RV and LV, and between the HLHS and wildtype samples.
